# Selections

**Published:** 1894-12

**Authors:** 


### Selections.

### CHEMICAL RESEARCHES ON THE MINERAL MATTER OF BONE AND TEETH.

Dr. S. Gabriel (*Ztschr. f. Physiol. Chem.*, xviii. p. 257) expresses the composition and properties of the ash of bone and teeth by the following general formula: $Ca_3(PO_4)_2 + Ca_5HP_3O_{13} + 2H_2O$, in which from two to three per cent. of calcium is replaced by magnesium, potassium, and sodium, and from four to six per cent. of phosphoric acid is replaced by carbonic acid, chlorine, and fluorine. The quantity of chlorine amounts to only a few hundredths of one per cent., except in the enamel of the teeth, which contains a relatively larger quantity (0.21 per cent.). Fluorine is a constituent of both bone and teeth. The quantity does not usually exceed 0.05 per cent. of the ash, rarely amounting to 0.1 per cent. In direct contrast to the other solid tissues of the body, bone and teeth contain more sodium than potassium. While the quantities of calcium and phosphoric acid vary only inconsiderably, yet the magnesium and carbonic acid vary inversely with these, so that the sums of the two bases and of the two acids are constant. The distinctive character of the ash of particular bones depends on the variation in the amount of calcium and phosphoric acid replaced, and also on the nature of the replacing radicles. The difference that exists between bone-ash and tooth-ash is not greater than the difference which has been observed in the ash of different bones. In enamel there is less, and in the body of the tooth relatively more, calcium replaced by magnesium than in bone-ash.

As the formula indicates, the phosphate is basic in character and is probably a combination of a neutral and a basic phosphate. The relative valence of the acid and basic radicles is as 15 to 16. Water is present in two combinations,—water of crystallization, driven off at from 300° to 350° C., and water in chemical combination, driven off at white heat when the substance is mixed with silicic acid.—*New York Medical Journal.*

